# Adapted Design Process for Continuous Fiber-Reinforced Additive Manufacturing—A Methodological Framework

**DOI:** 10.3390/ma17133194

**Published:** 2024-06-29

**Authors:** Tim Heitkamp, Karl Hilbig, Simon Girnth, Sebastian Kuschmitz, Nils Waldt, Günter Klawitter, Thomas Vietor

**Affiliations:** 1 Institute for Engineering Design, Technische Universität Braunschweig, 38108 Braunschweig, Germany; 2 Faculty II, Hochschule Hannover, University of Applied Sciences and Arts, 30459 Hannover, Germany

**Keywords:** design for additive manufacturing, DfAM, industrial product design, product development, continuous fiber, fiber-reinforced additive manufacturing

## Abstract

Continuous fiber-reinforced material extrusion is an emerging additive manufacturing process that builds components layer by layer by extruding a continuous fiber-reinforced thermoplastic strand. This novel manufacturing process combines the benefits of additive manufacturing with the mechanical properties and lightweight potential of composite materials, making it a promising approach for creating high-strength end products. The field of design for additive manufacturing has developed to provide suitable methods and tools for such emerging processes. However, continuous fiber-reinforced material extrusion, as a relatively new technology, has not been extensively explored in this context. Designing components for this process requires considering both restrictive and opportunistic aspects, such as extreme anisotropy and opportunities for functional integration. Existing process models and methods do not adequately address these specific needs. To bridge this gap, a tailored methodology for designing continuous fiber-reinforced material extrusion is proposed, building on established process models. This includes developing process-specific methods and integrating them into the process model, such as a process selection analysis to assess the suitability of the method and a decision model for selecting the process for highly stressed components. Additionally, a detailed design process tailored to continuous fiber-reinforced material extrusion is presented. The application of the developed process model is demonstrated through a case study.

## 1. Introduction

Additive manufacturing (AM) is a collective term for a variety of generative manufacturing processes characterized by a layer-by-layer building principle [1,2]. Initially, additive manufacturing was primarily utilized for the cost-effective and rapid production of demonstration objects and prototypes. However, these processes are progressively gaining importance for manufacturing end products [1,3]. The main advantages of additive manufacturing processes include great design freedom combined with a high degree of customization, the economical production of small quantities, and potential for component consolidation or functional integration [3,4,5]. However, venturing into new application areas also involves addressing increasing mechanical and functional requirements, which must be tackled from both process engineering and design engineering perspectives. A significant factor impeding the further dissemination and substitution of conventional manufacturing processes by additive processes is the lack of available knowledge and methods that support manufacturing-oriented design and the utilization of unique process-specific potentials [6,7,8,9]. To counteract this deficiency, methods and tools designed specifically for additive manufacturing are being developed in the research field of design for additive manufacturing (DfAM). One process that has received inadequate attention in this context is continuous fiber-reinforced material extrusion (CFR-MEX), which is a relatively young manufacturing technology compared to established additive manufacturing processes, characterized by unique lightweight potentials with load-appropriate fiber alignment and a high potential for functional integration [10,11,12,13,14]. Moreover, CFR-MEX presents several significant restrictions and peculiarities compared to other additive manufacturing processes. Therefore, methods for the early assessment of CFR-MEX’s potential for the given design tasks are lacking. Additionally, there is neither an adapted design process nor a model that accounts for the extreme anisotropy within the product development process. In addition, previous work [15] has developed process-specific design knowledge, which should be integrated with product development phases for comprehensive utilization. This paper proposes a comprehensive framework to address these research gaps.

In the following section, CFR-MEX is first presented, followed by the previous efforts in the research field of DfAM. The objective and the research gap to be addressed by this article are then presented in Section 4. Building upon the identified research gap, the framework and the embedded DfAM tools are discussed in Section 5. This is followed by an exemplary demonstration of the framework. The article concludes with a section that also includes an outlook on future possible research projects.

## 2. Continuous Fiber-Reinforced MEX

**Figure 1:**
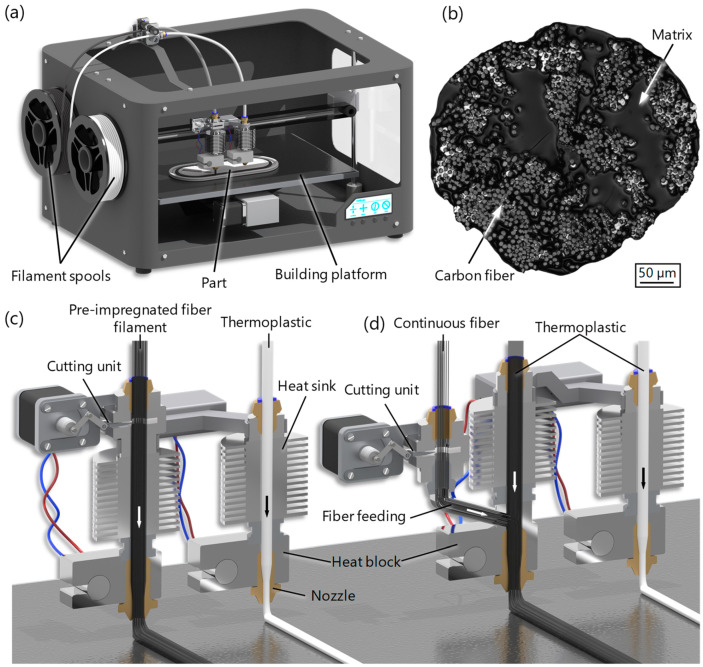
Schematic representation of the CFR-MEX manufacturing process: (**a**) printer system [41]; (**b**) light microscope image of the cross-section of the pre-impregnated continuous carbon fiber filament [43]; CFR-MEX approaches (**c**) using pre-impregnated fiber filament; (**d**) merging and impregnating fibers within the printhead [41].

Conventional MEX [16] (also known as FDM—fused deposition modeling—or FFF—fused filament fabrication) is characterized by a process principle in which a three-dimensional component is produced by the layer-by-layer deposition of a thermoplastic strand [1,3]. MEX has so far been used predominantly for the production of demonstration objects and prototypes. Since the mechanical properties of components produced using MEX often do not meet the requirements for mechanically stressed end products, the process is rarely considered when selecting suitable manufacturing processes [17,18]. A proven method for reinforcing plastic components is the addition of reinforcing fibers. While short fiber reinforcement (0.1–1 mm) results in only a slight improvement in mechanical properties, continuous fiber reinforcement leads to a significant increase in mechanical properties [19,20]. So far, two different approaches have been established, which differ in the timing of fiber impregnation (Figure 1). In one approach, the continuous fibers are already impregnated with a thermoplastic matrix in the form of an extrudable filament—the so-called pre-impregnated fiber filament (Figure 1c) [21]. In the second approach, a fiber roving, which may have been treated with a sizing agent, is fed into the print head. Only then are the fibers combined with the molten thermoplastic in the print head and subsequently deposited onto the print bed (Figure 1d) [22,23,24,25]. A cutting unit within the print head allows for the deposition of multiple independent fiber strands in the layer plane. Carbon, glass, or aramid fibers are predominantly used as reinforcing fibers [26,27,28].

The layer-by-layer principle underlying additive manufacturing leads to anisotropy in additively manufactured components, with lower strengths and stiffnesses in the build direction compared to the strengths and stiffnesses in the layer plane [29,30]. This anisotropy is greatly amplified by the layer-by-layer incorporation of continuous fibers. This allows the mechanical properties to be significantly increased in the layer plane. In the build-up direction, however, the mechanical properties of the plastic can even be reduced by the continuous fiber embedding. As a result, using CFR-MEX instead of conventional MEX does not necessarily lead to an improvement in mechanical properties if the component is not oriented appropriately or if loads occur in multiple spatial directions. In addition to the build-space orientation, the alignment of the fibers is also crucial. A load-appropriate fiber alignment can significantly increase mechanical properties, meaning that fill patterns must also be given increased attention compared to conventional additive manufacturing processes. Therefore, a large part of the research in the field of DfAM focuses on methods for optimizing fiber alignment [31,32,33,34,35,36]. Although continuous fibers are primarily used to improve mechanical properties, their use also offers enormous potential for functional integration. For example, the introduction of fibers allows for the integration of electrical circuits [37,38,39], sensory properties [10,11,12,40,41], or electromagnetic shielding [42] into components. A comprehensive overview of the numerous design potentials and also the manufacturing restrictions are described in detail in [15].

## 3. Design for Additive Manufacturing

The roots of the research field of DfAM are grounded in established approaches such as design for manufacturing (DFM) and design for assembly (DFA) [3,44]. However, there is currently no universally accepted definition or standardized term for this concept [6]. Rosen [45] summarizes DfAM as the “synthesis of shapes, sizes, geometric mesostructures, and material compositions and microstructures to best utilize manufacturing process capabilities to achieve desired performance and other life-cycle objectives”. Expanding on this, Gibson et al. [3] attribute to DfAM the goal of enhancing the performance of components manufactured using additive manufacturing. Laverne et al. [7,46] ascribe to DfAM all methods and tools that aid the design process by considering process-specific peculiarities. In recent years, there has been intensive research into adapting models for the development and design of components and systems to be produced using additive manufacturing [47]. Ponche et al. [48,49] developed a DfAM procedural model specifically for the manufacture of thin-walled metal components using direct energy deposition (DED). Yang and Zhao [50] also developed a procedure model with a focus on functional integration and structural optimization. However, their model aimed at optimizing existing components that were designed for conventional processes. Kumke et al. [9] proposed a methodological procedural model for developing components manufactured via additive manufacturing based on VDI Guideline 2221 [51], a procedural model widely adopted in German-speaking regions. Key changes in this model include a decision node after the conceptual phase to consider the technical and economic potentials and limitations of additive manufacturing methods and to evaluate their suitability for the product to be developed. Moreover, tools are specifically integrated into the framework to promote the extensive utilization of AM-specific potentials. A comprehensive framework for the development of additive manufactured components was also presented by Zhu et al. [52]. The framework was developed based on surveys from experienced DfAM designers. In contrast to the approach of Kumke et al. [9], this method prioritizes process selection before the conceptual phase. This early process selection allows for the exploitation of all AM-optimized phases of the framework, starting with the conceptual phase. Both Kumke et al. [9] and Zhu et al. [52] assign path planning, build-space orientation, and fill pattern design to the manufacturing responsibility in their procedure models. Kranz et al. [53] also introduced a procedural model based on VDI Guideline 2221 [51]. The model’s focus lies on developing lightweight design through the utilization of the laser powder bed fusion (LPBF) process. CFR-MEX has not yet been considered in the context of adapted product development processes in previous studies. The research gaps that need to be addressed are discussed in the following section.

## 4. Research Gap and Objective

In the previous section, the numerous efforts in the context of DfAM with regard to the development of adapted product development processes were discussed. Despite significant research in recent years within the DfAM field, CFR-MEX has not received substantial attention regarding methodological support for the development process. However, CFR-MEX occupies a special position among additive manufacturing processes. Despite its relatively low cost, the technology can manufacture components capable of enduring substantial mechanical stress—a characteristic highly pertinent to future lightweight design applications. Thus far, CFR-MEX has primarily been employed when mechanical requirements cannot be met by unreinforced plastic-based components. Often, the diverse opportunities for functional integration are disregarded. Simultaneously, specific features must be considered during the development and design of components intended for CFR-MEX manufacturing. These include, on the one hand, the strong anisotropy, which by far exceeds the usual anisotropy for AM processes due to the layer-by-layer process principle. In addition, previous process models lack methods for the early selection or limitation of the manufacturing process in order to avoid undesirable developments and to limit the design knowledge to be taken into account. Furthermore, production planning is of particular importance in comparison with other additive processes. Due to the integration of continuous fibers, manufacturing parameters such as the orientation of the build chamber and the alignment of the continuous fibers in the layers are of decisive importance in the development of components subject to high mechanical loads. Thus, this paper will address the key limitations of current approaches and models, encompassing the following aspects:

- The design of components and systems to be manufactured using CFR-MEX should be embedded in the development process and methodically supported.
- The focus of conventional process models and also AM-adapted methods is often on the mechanical properties or the realization of a specific AM potential; this leaves little room for the integration of additional functions and the exploitation of all process-specific potentials.
- To date, no process-specific decision support tools have been developed for process selection and evaluation to ensure process suitability for the product being developed.
- Due to the significant effects of fiber orientation, production planning has a considerable influence on the resulting properties. However, in existing development models, the filling pattern generation of the components to be additively manufactured is not part of the design process but is attributed to the manufacturing experts.

The aim of this work is therefore to develop a holistic approach in the form of a process model for the development and design of components to be manufactured using CFR-MEX. For this purpose, a framework model is proposed, which fully considers the aspects mentioned above. Process-specific DfAM tools in the form of a process selection analysis, an adapted design process, and a decision model are linked to the framework model.

## 5. Design Framework

The previous section highlighted the necessity for an adapted process model tailored to CFR-MEX. The adapted process model is based on the established model from VDI Guideline 2221 [51], while also considering the development methodology outlined in VDI/VDE Guideline 2206 [54]. The process model according to VDI Guideline 2221 [51] has already been proposed by Beyer [55] for AM and adapted by Kumke et al. [9] for general AM. Utilizing an established and widely accepted process model also enhances its acceptance and simplifies its application for experienced designers. The development or adaptation of the process model is based on the limitations of existing approaches and fundamental requirements for process models for the development of technical products, as outlined in the previous section.

### 5.1. Requirements for the Process Model

As a basis for adapting the process model, requirements are formulated. In total, the following seven requirements of a “general”, “AM-specific”, and “CFR-MEX-specific” nature were established (Table 1). The assignment of the requirements to these categories is intended to facilitate future adaptation of the developed process model to other additive manufacturing processes or further developments in CFR-MEX.

General requirements are basic requirements for process models for the development and design of technical components, while AM-specific requirements apply to AM regardless of the specific process. These requirements should also be considered in any future adaptations of the process model, independent of the specific AM process. On the other hand, process-specific requirements relate only to the particularities of CFR-MEX and may not be relevant for other AM processes.

### 5.2. Adapted Process Model

**Figure 2:**
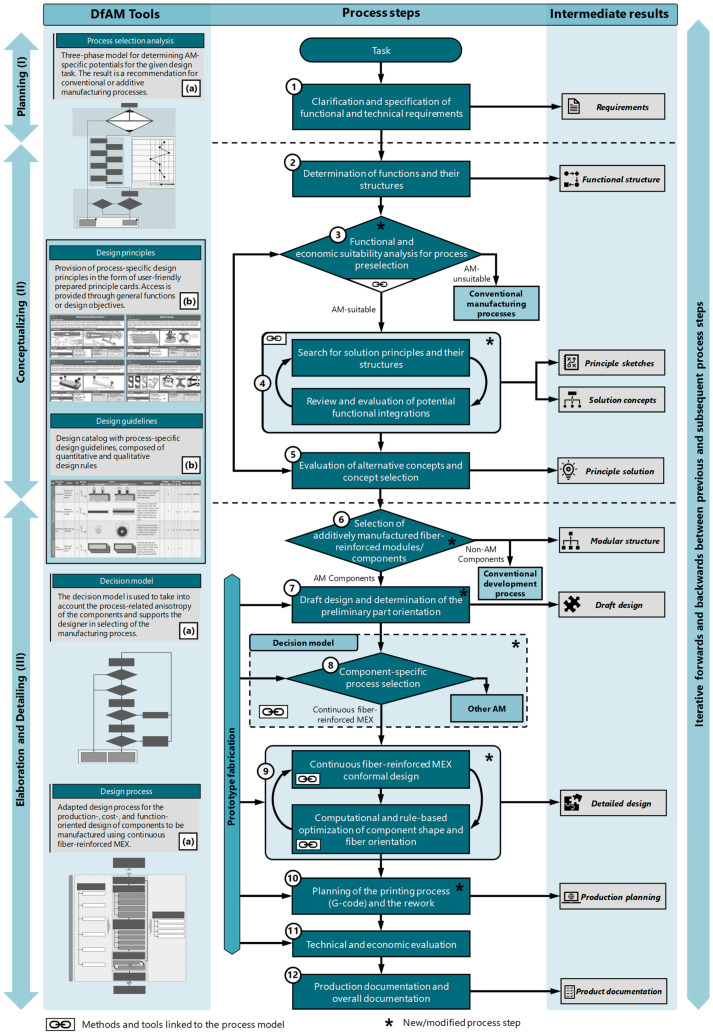
Adapted process model for CFR-MEX with linked DfAM tools: (**a**) provided within the scope of this work, (**b**) provision of process-specific design knowledge [15].

Based on the established requirements, the adapted process model results in a three-stage phase model, consisting of the phases “planning (I)”, “conceptualizing (II)”, and “elaboration and detailing (III)”, with twelve process steps (Figure 2). Analogous to VDI Guideline 2221 [51], the process steps can be skipped, repeated, or carried out simultaneously as needed. The process model supports the “coarse-to-fine” approach, with continuous product concretization as the development process progresses. In phases I and III, decision nodes are implemented and associated detailed selection processes are proposed, which relate to the process selection or limitation. A total of ten intermediate results are assigned to the process steps, which are based on the results of VDI Guideline 2221 [51] and enforce the basic “coarse-to-fine” approach (Figure 2). The adapted process model is designed as a flowchart and consists of three columns. In addition to the centrally arranged process steps, the intermediate results of the respective process steps are noted on the right side. On the left side, prepared DfAM tools are linked with the corresponding phases. These include restrictive and opportunistic design knowledge [15], a potential-based process selection analysis (Section 5.3), and a decision model for considering mechanical stresses (Section 5.4). The individual phases and associated process steps are described in more detail below.

#### 5.2.1. Planning Phase

Analogous to VDI Guideline 2221 [51], a detailed task description, followed by the clarification and specification of functional and technical requirements, forms the basis for product development. The product requirements are collected in a requirement list, which is continuously updated. The collection of requirements is independent of the type of product development, i.e., for new designs as well as for adaptation and variant designs.

#### 5.2.2. Conceptualization Phase

Based on the requirement list, the determination of functions and their structures is carried out in the first step of the concept phase, analogous to VDI Guideline 2221 [51]. This involves decomposing the overall function(s) of the component/system to be fulfilled into individual sub-functions and describing their relationships with each other. 

Eddy et al. [56] and Pradel et al. [47] suggested promoting process selection as early as possible in the development process to prevent undesirable developments. Therefore, a decision step follows the functional structure, in which an initial process preselection or a limitation of the considered processes takes place. An initial preselection can be based on a variety of factors, such as quantities and costs, development duration, desired materials, system or component size, target weight, equipment availability, and mechanical properties. To support decision making, a process selection analysis was developed, which is described in detail in Section 5.3. The result of this process selection analysis is the decision as to whether CFR-MEX or AM in general should be considered at all in the further development process or whether the product is not suitable for AM. The early inclusion of suitable (additive) processes also enables a comprehensive and early consideration of process-specific potentials in the search for solution principles in the subsequent process step. It should be noted that this can also lead to pre-fixation, highlighting the importance of the ongoing evaluation of alternative manufacturing methods. Furthermore, it should be acknowledged that an early preselection of suitable manufacturing processes for a product or component, especially in the case of new developments, is not always feasible. Building on this, suitable solution principles can be sought for the established sub-functions in an iterative approach in the subsequent step, and potentials for functional integration can be identified. In this context, process-specific design principles or heuristics, for example, are particularly important as DfAM tools, which help in considering the process-specific potentials and support the solution search according to needs [15,57,58,59]. 

Nevertheless, the deliberate application of opportunistic design knowledge assumes an initial process preselection or restriction, underscoring the significance of early process evaluation. Based on the developed solution concepts, the concept evaluation and selection are finally carried out. A renewed suitability analysis of the process selection can also take place in the course of the concept evaluation.

#### 5.2.3. Elaboration and Detailing Phase

In the elaboration and detailing phase, the product/component is designed and detailed. During phase III, the option of producing prototypes can be explored at different junctures. The manufacturing of prototypes can serve for functional evaluation, geometric verification for assembly purposes, or for checking the aesthetic impression [2]. Based on the established solution concept in phase II, the components to be produced using additive manufacturing are first selected and considered in the further course of the process model. This is followed by an initial draft design of the selected components, along with a preliminary build-space orientation. The early determination of the build-space orientation is generally a central process step for additive manufacturing processes, as the layer-by-layer building principle inevitably results in anisotropic component properties. In the case of CFR-MEX, however, this circumstance has a special significance compared to other processes, as the anisotropy is much more pronounced [60,61]. Moreover, the draft design and build-space orientation are used in the following step to perform a final process selection based on these features. For mechanically highly stressed components, a decision model has been implemented in the process model (described in detail in Section 5.4). The aim of this model is to support the user in making a decision regarding the process to be used. The result of this decision model is the final decision for or against CFR-MEX. If a decision is made not to use CFR-MEX, conventional development processes should be used. For the components to be produced using CFR-MEX, the design and detailing of the components are carried out in the following process step. To support the challenging design process, a process-specific design process was developed (described in detail in Section 5.5). The result of the developed design process is the detailed design of the component. Based on the detailed design, production planning is carried out (G-Code—a machine code readable by 3D printers [62]). In contrast to existing general and AM-specific process models, this process step is understood as an elementary part of the development process. Despite its thematic affiliation with manufacturing, path planning is of crucial importance for the resulting mechanical properties of the component, as they depend in particular on fiber orientation, fiber volume content, and cutting positions of the continuous fiber strands. This is followed by a technical-economic evaluation of the developed solution, which may also include alternative solutions using other manufacturing processes. The process model concludes with the preparation of manufacturing documents and overall documentation.

### 5.3. Process Selection Analysis

**Figure 3:**
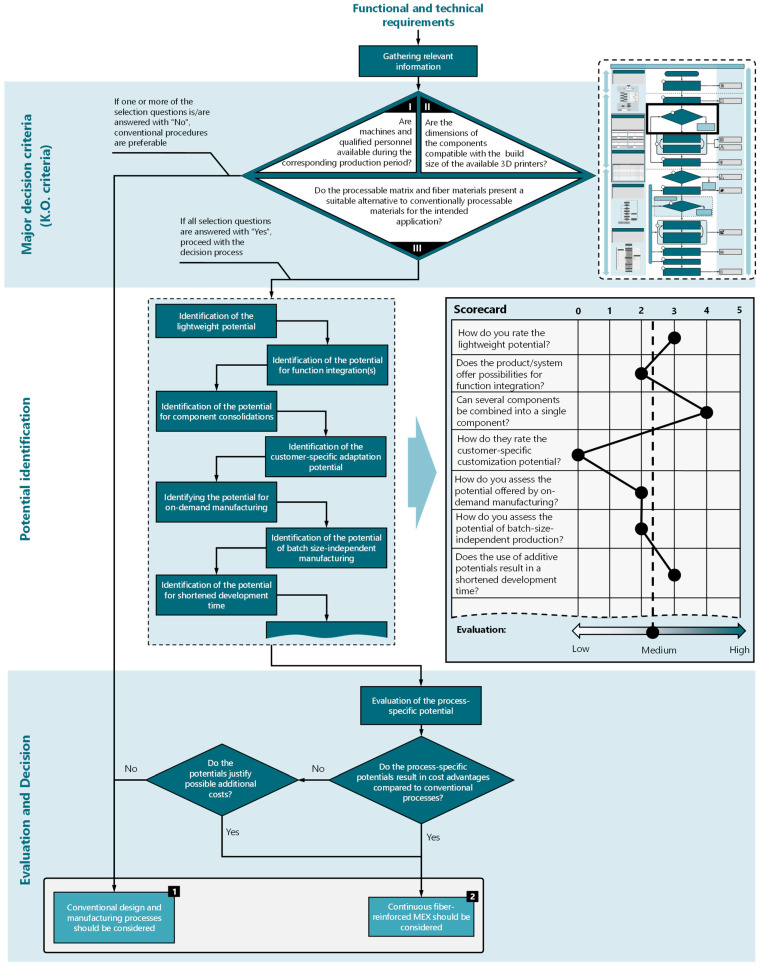
Potential model for the process evaluation of CFR-MEX and additive manufacturing processes for the planning phase of the adapted process model, with an exemplary potential assessment based on an unweighted point evaluation.

Selecting manufacturing processes early enhances the utilization of process-specific potentials and ensures the timely consideration of these potentials and any restrictive limitations [47,52,56]. However, decision making often depends on subjective assessments and personal experience. Therefore, a potential-based model is implemented in the process model, which supports designers in decision making (Figure 3). The potential model is divided into three phases: “major decision criteria”, “potential identification”, and “evaluation and decision”. The preceding process step in the process model (Figure 2) provides for the collection of functional and technical requirements for the product. From there, the first step is to collect all relevant information about the product/component to be developed. This is followed by a decision node consisting of three knockout criteria. Negating one of the knockout criteria results in the exclusion of AM/CFR-MEX. The decision criteria are composed of the following questions:

- Can the required quantities be realized using AM within the planned production period?
- Are the anticipated component dimensions manufacturable with the available equipment?
- Do the processable (matrix and fiber) materials represent an alternative to conventionally processable materials?

With regard to the choice of matrix and fibers, it should be noted that the specific materials that can be processed using continuous fiber-reinforced MEX are sold by the respective suppliers of printer systems. This ensures that the materials are always compatible with the printer system used. At the same time, the choice of matrix and fiber materials is limited.

If the three criteria listed do not lead to exclusion, the next step is to identify potential. The focus of potential identification is on determining usable process-specific potentials that, compared to conventional processes, can bring about improvements in product quality, sustainability, production, usage, and storage costs, or even shorten development time. However, the potentials listed in Figure 3 are not exhaustive and may vary depending on the specific process. An evaluation of the potentials can be carried out using a weighted or unweighted point evaluation, as schematically shown in Figure 3. Other evaluation methods, such as utility analyses, checklists, or preference matrices, are also conceivable.

Following the evaluation, a decision node is proposed, which provides a cost estimate for conventional manufacturing and AM, taking into account the usable potentials. If a cost advantage arises from using AM, it should be preferred over conventional manufacturing. If additional costs are incurred, a subsequent process step should assess whether these costs are justified by significant improvements, such as product quality, increased customer benefits, or aesthetic factors.

### 5.4. Decision Model

**Figure 4:**
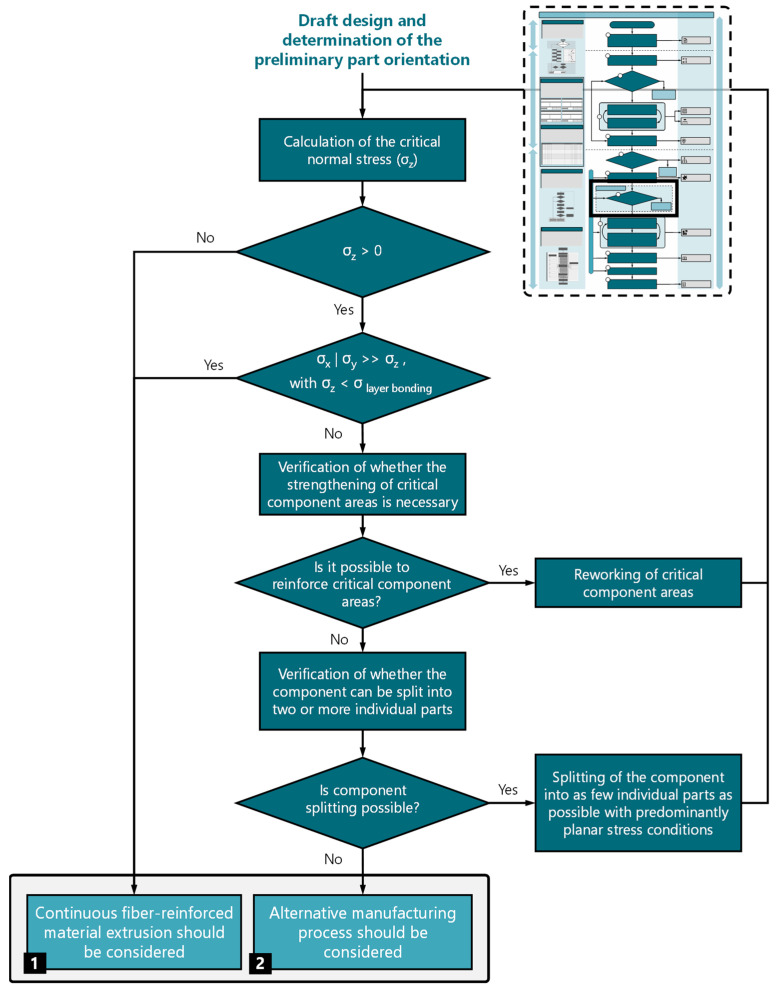
Decision model for mechanically heavily loaded structural components to evaluate the suitability of the manufacturing process.

After selecting the components to be additively manufactured and creating the draft design, a decision model (Figure 4) has been implemented in the process model for mechanically highly stressed components. The model takes into account the extreme anisotropy of fiber-reinforced printed components due to the process and supports the designer in selecting the process. The decision regarding CFR-MEX, for instance, considering functional integrations, should be evaluated separately from this process.

The model (Figure 4) is designed as a flowchart and starts with an initial analysis of the occurring loads, followed by a decision node. If loads only occur in the layer plane, CFR-MEX should be considered as the manufacturing process (Case 1). If significant loads are present in the direction of the layer normal, the subsequent step involves determining the dominant load plane. If the load dominance occurs within a spatial plane, the next step involves calculating the loads in the layer normal direction for the less stressed spatial direction, based on the draft design. Subsequently, an assessment is conducted to determine if the interlayer bond strength is adequate for the current load case. If the bond strength is sufficient for the load case, CFR-MEX can also be considered (Case 1). If significant loads occur in several spatial directions or if the bond strength is insufficient, it should be checked whether the critical component areas can be reinforced. If this is not possible, splitting the component into two or more parts can be considered. However, one must be aware that this approach gives up a significant advantage of additive manufacturing processes. If splitting the part is not an option for the current design, it is advisable to investigate alternative manufacturing processes (Case 2).

### 5.5. Adapted Design Process

**Figure 5:**
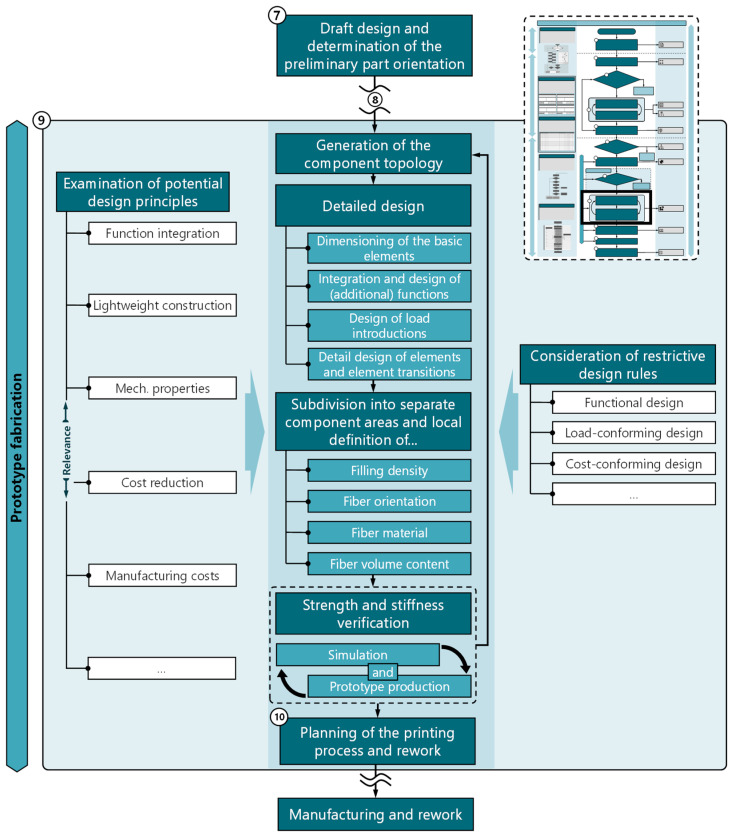
Design process tailored to the CFR-MEX with linked design knowledge (prepared and provided in [15]).

The design process plays a central role in the development of products and systems. In particular, for processes with novel potentials and restrictions, a design process tailored to the specific requirements of the process is crucial and can make a significant contribution to the development outcome. The design process (Step 9) follows the initial draft design (Step 7) and, for mechanically highly stressed components, the subsequent decision for or against the use of the process (Step 8). The approach model (Figure 2) divides the design process into an iterative procedure consisting of the two process steps “designing the component for manufacturing and functional requirements” and “computer- and rule-based optimization of component shape and fiber orientation”. A more detailed flowchart for the adapted design process is shown in Figure 5. 

Throughout the design process, it is essential to integrate process-specific design knowledge to ensure a functional, manufacturable, and appropriately functional design while fully capitalizing on all potentials. Based on the rough design of the component, the component topology is first generated. The component topology can be generated for less complex components on an intuitive basis and for complex components using simulation-supported optimization methods [63]. The detailed design, consisting of four individual steps, follows the component topology: first, based on the topology, the basic elements are designed, considering the existing process restrictions (e.g., minimum wall thicknesses, hole diameters, minimum pin diameters, etc.). This step is followed by the integration and design of (additional) functions. Since the integration of additional functions may involve significant changes in the component geometry and system architecture, the integration of additional functions should take place after the design of the basic elements. For fiber-reinforced components subject to high mechanical stresses, the next step is to design the load introduction into the component. Experimental verification of the design solution should always be performed, since the accuracy of computer-aided modeling is often insufficient [64]. The subsequent detail design of elements and element transitions should already be carried out with a view to a load- and function-appropriate fiber alignment and also to minimize the need for support structures. 

The next process step involves dividing the component into meaningful areas. The separate consideration of components aims to support local adjustment of fill density, fiber orientation, fiber material, and fiber volume content to the mechanical stresses occurring in the respective areas. This allows for effective savings in material and weight as well as manufacturing time and associated costs.

Based on the developed component shape, a strength and stiffness verification is carried out using simulation-based methods. Alternatively, or additionally, a prototype can be manufactured with experimental strength and stiffness verification. This is particularly useful for components with complex fiber layouts, the simulation of which can be inaccurate and complex. If the simulation or experimental investigations yield satisfactory results, the printing process and any rework are planned. Insufficient strength and stiffness can be addressed by readjusting the preceding process steps.

### 5.6. Adaptation and Variant Designs

**Figure 6:**
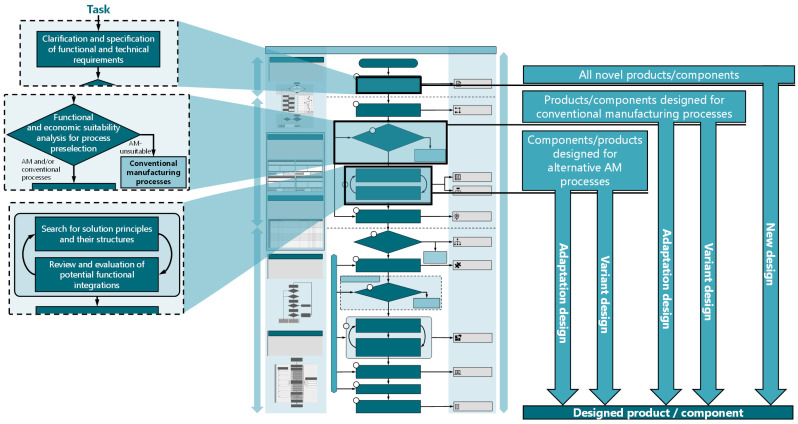
Initial process steps for novel, adaptation, and variant designs in the process model for CFR-MEX.

The main focus of the adapted process model is on innovative new designs. This emphasis is reflected in its foundation on a comprehensive approach encompassing the entire development process, commencing with the identification of requirements. However, in industrial practice, a large part of developments involves adaptation or variant designs [65,66]. Figure 6 presents a schematic representation of the initial process steps for novel, adaptation, and variant designs within the adapted approach model. 

According to Pahl and Beitz [66], adaptation designs adhere to existing solution principles and merely adapt the design to new boundary conditions. In adaptation designs, established principles of action remain, while the focus is on redesigning details. For variant designs, usually only the dimensions and arrangements of design elements are changed [67]. However, this approach is only partially suitable for AM, as components designed for conventional manufacturing processes may be completely unsuitable for AM [68]. Components developed for conventional processes may not be producible using AM or may have an uneconomical component design for AM. Moreover, directly transferring component designs from conventionally manufactured parts to additively manufactured counterparts leads to the inadequate exploitation of process-specific potentials. Therefore, a process selection step must be implemented for adaptation and variant designs to ensure their general suitability for AM. Components and systems that have previously been produced using AM constitute an exception to this rule. As a result, the adapted procedural model for adaptation and variant designs is segmented into products/components tailored to conventional manufacturing processes and those already engineered for additive manufacturing (Figure 6).

Regardless of the type of design, the design process begins with the modification and adaptation of existing requirements. To fully exploit the potential of process-specific capabilities, it is necessary to repeat or revise steps from the planning or concept phase in adaptation and variant designs as well. For products/components designed for conventional manufacturing processes, a functional and economic suitability analysis is a sensible starting point to ensure that AM is suitable for the product/component. For products/components already developed for AM, the evidence of basic suitability for AM has already been provided. However, since the solution principles and possibilities for function integration vary greatly between additive processes, the development should begin with the search for solution principles and possibilities for function integration.

## 6. Case Study

To validate and illustrate the developed procedural model, the following section presents a case study that exemplifies the development process. Prior to this, the methodological approach was applied to various components and products, such as a bicycle crank [15,43] and functional drone arms, to verify and improve its applicability to different product contexts. In the following, however, the procedural model will be demonstrated using a protective mask for athletes following a facial fracture, such as a nasal bone fracture. These protective masks serve to shorten break times and prevent re-fracturing during sports activities [69,70]. The following figures depict the exemplary development phases, providing explanations of the corresponding process steps and interim outcomes. The focus is on the process steps deviating from the underlying process model. 

**Figure 7:**
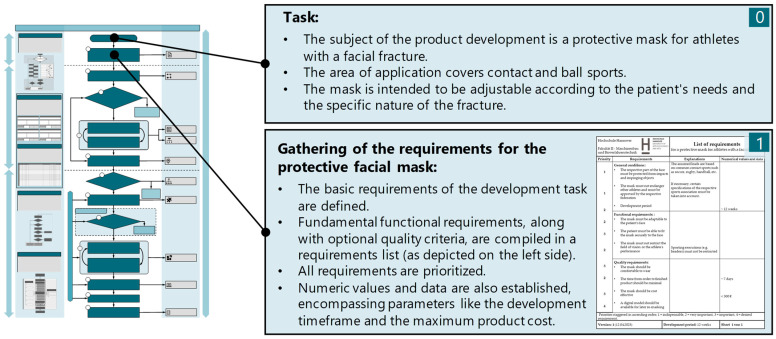
Planning phase (phase I).

Given that this is a novel development, the process model is fully implemented, commencing with the task definition and the compilation of a requirements list. The requirements list compiles the fundamental framework conditions for specific product development, in addition to the functional and quality criteria imposed on the mask. The list of requirements is continuously adapted and revised throughout the product development process (Figure 7).

**Figure 8:**
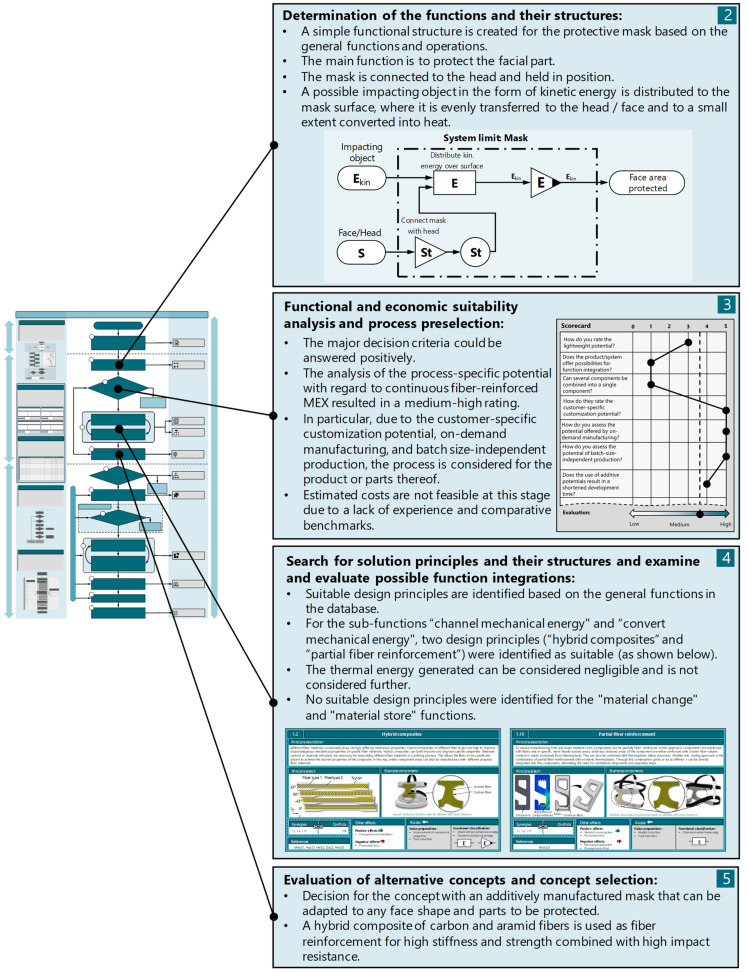
Concept phase (phase II).

The conception phase, shown schematically in Figure 8, begins with the determination of functions and their structures. To enhance clarity, the functional structure has been restricted to include only the primary functions. The main functions include, on the one hand, attaching the mask to the athlete’s head to cover the facial areas to be protected. On the other hand, a primary function is the protection of the corresponding facial area from an impacting object. This protection is ensured by the absorption of mechanical energy and its distribution across the surface of the mask, followed by even distribution on the athlete’s face. After creating the functional structure, the process selection analysis is carried out to determine the suitability of the product for general additive manufacturing or CFR-MEX. The suitability analysis reveals a medium to high potential for additive manufacturing (and CFR-MEX), primarily resulting from the process-specific potentials for customer-specific adaptations, demand-driven production, and batch-size-independent manufacturing. A cost estimate is not yet possible due to the lack of comparable components. 

Based on the functional structure, suitable solution principles for the individual sub-functions are identified. A knowledge system for CFR-MEX [15] is used for this purpose. With the help of this knowledge system, two suitable principles for the function “conduct mechanical energy” were identified. First, “partial fiber reinforcement” was identified. This principle involves reinforcing only specific highly stressed fiber areas, or the areas to be protected by the mask, with continuous fibers. This leads to lower costs and increased manufacturing speed. In addition, the remaining areas of the mask can be made from unreinforced flexible thermoplastic, which improves adaptation to the facial shape and increases wearing comfort. The second principle selected was “hybrid composites”. Since the facial areas to be protected require a material with both high strength and stiffness but also require high impact toughness, carbon fibers alone may be unsuitable as reinforcement material. The principle involves mixing different fibers, such as carbon and aramid fibers, to specifically adjust the mechanical properties of the components. No suitable principles were identified in the database for the remaining functions. The use of straps is intended for attaching the mask. The creation of additional concepts was omitted. 

**Figure 9:**
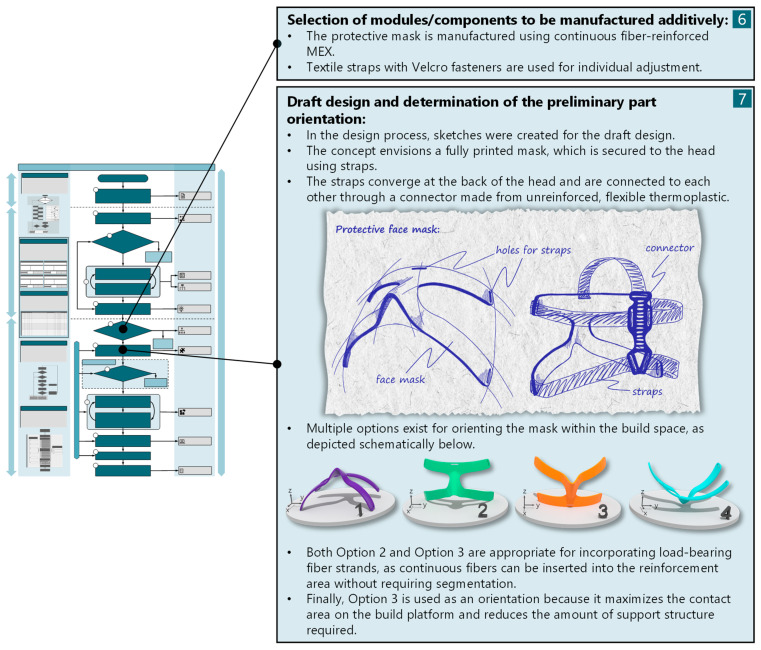
First two process steps of the elaboration and detail design phase (phase III).

The elaboration and detail phase starts with the selection of the components to be manufactured additively. The chosen concept proposes that both the mask itself and the connector for securing the straps should be additively manufactured. Other components of the mask, such as straps, padding, and Velcro fasteners, will be procured. Subsequently, an initial draft design of the mask and the assembly is created. Based on the concept sketches, an initial 3D model is created, in which the possible build-space orientations are schematically illustrated (Figure 9). 

Various options are available for orientation within the build space. For a load-appropriate fiber insertion, options 2 and 3 can be considered. In this case, the fibers can be printed in a continuous form without interruptions in the nasal area to be protected. Ultimately, option 3 was selected, as it minimizes the amount of necessary support structure and maximizes the contact surface between the mask and the build platform (Figure 9).

**Figure 10:**
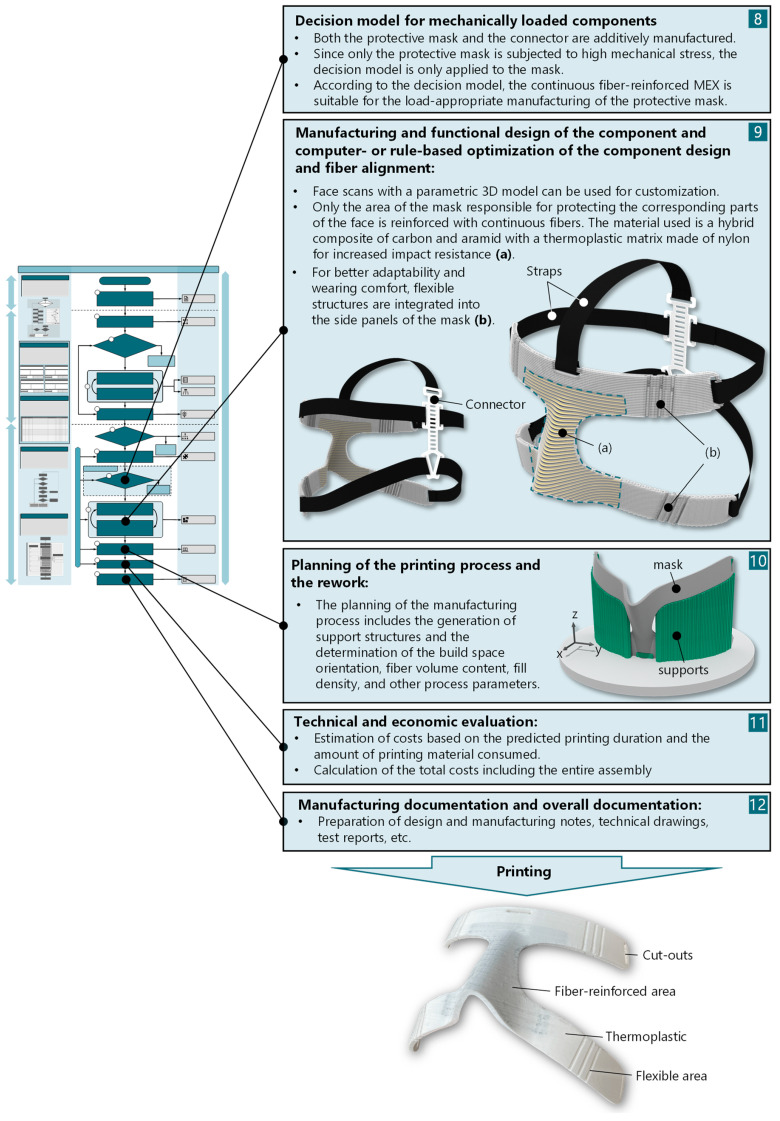
Remaining process steps of the elaboration and detail design phase (phase III).

Since only the mask itself is manufactured using CFR-MEX, the decision model for mechanically highly stressed components is applied. After deciding to use the CFR-MEX for the protective mask, the detailed design of the mask is carried out (Figure 10). 

For an optimal fit, facial scans can be used to generate custom-fit masks using a parametric 3D model. For the demonstration, the creation of a parametric model was omitted. In accordance with the identified design principles [15], only the component areas relevant to the facial parts to be protected are reinforced with continuous fibers. To improve the impact toughness of the carbon fiber-reinforced composite material, a hybridization of carbon and aramid fibers is used. In addition, elastic structures are integrated into the side parts of the masks to improve wearing comfort and adaptability. A technical-economic evaluation can be carried out based on the predicted printing time and the amount of material consumed, as well as the procured standard components. Finally, all documentation for manufacturing, distribution, certification, recycling, and disposal is created. Some specifications of the printed mask are listed in Table 2. The mask was produced on a Markforged X3 (Markforged, Inc., Watertown, NY, USA). The specifications were calculated using the Markforged slicing software (Eiger – cloud version) [71]. Since the printer only has a print head that can extrude continuous fiber-reinforced thermoplastic, the mask was only reinforced with carbon fibers.

## 7. Conclusions

In this paper, a methodical approach model for the development and design of additively manufactured composite components is presented, which is based on the approach model in VDI Guideline 2221 [51]. The main limitations of previous design methods concerning CFR-MEX include the complex design process, which requires a multitude of special features and process-specific knowledge to ensure optimal component design and the comprehensive utilization of process-specific potentials. Furthermore, the majority of existing approach models focus on a specific AM potential. Additionally, a deficiency exists in terms of process-specific DfAM tools, including tools for early process evaluation and selection, as well as methods for optimizing build-space orientation. These limitations were formulated as requirements, based on which the approach model was adapted. The result of this development is an approach model divided into three phases with a total of twelve process steps. In addition to adapted process steps, a potential analysis, a decision model, and an adapted design process were implemented in the approach model. The potential analysis serves to determine the suitability of the process early in the product development process. The decision model factors in the significant anisotropy present in fiber-reinforced printed components due to the manufacturing process. This model aids designers in the selection of the appropriate manufacturing process. Furthermore, manufacturing planning concerning the orientation in the build space and the determination of fiber alignment, fiber volume content, and fill density were attributed to the area of responsibility of the design due to their immense importance for product performance. However, the approach model was deliberately kept neutral and can therefore also be used for other AM processes. Even if the decision is made not to use AM/CFR-MEX, the conventional approach model outlined in VDI Guideline 2221 [51] can still be followed without interruption. At the same time, the modularity of the approach model allows for the direct linking of future DfAM methods with the modified process steps. The approach model was demonstrated and validated using a case study, in which a protective mask for athletes with a facial fracture was developed based on the presented model.

Future research projects should continue to focus on the development and provision of process-specific design knowledge and DfAM tools for CFR-MEX. Furthermore, based on the presented model, adaptations for other additive manufacturing processes that have been inadequately considered in previous research can be adjusted. It would be useful to add methods for selecting the most suitable additive manufacturing process and adapted design processes in each case. With increasing knowledge of material failure and the mechanical properties of printed composites, mathematical models and algorithms should be developed in the future to provide additional support for the design process and the decision model. Additionally, increased research should be conducted on automated support for build-space orientation and the generation of optimal fiber paths.

## Figures and Tables

**Table 1:** General and AM/CFR-MEX-specific requirements for the adapted process model.

No.	Requirement	Type
1.1	The process model should encompass all phases of the product development process, from clarifying requirements to creating the concept, on to detailing and designing the component.	General
1.2	The process model should be applicable to all types of design (novel, adaptation, and variant designs).	General
2.1	In line with a holistic approach, process-specific opportunistic and restrictive design knowledge for additive manufacturing processes should be linked with the phases/process steps.	AM-specific
2.2	The development process must be suitable for conventional methods as well as for additive manufacturing/CFR-MEX until the final decision for using AM is made and should be designed as neutrally as possible. This allows individual modules/components to be manufactured conventionally, avoiding elaborate iterations.	AM-specific
2.3	To narrow down the design freedoms and potentials of the additive manufacturing process, an early selection of the process should be performed.	AM-specific
3.1	The build-space orientation should be determined early on, taking into account the extreme anisotropy in mechanically heavily loaded components, and critically assessed in conjunction with a suitability test of the manufacturing process.	CFR-MEX-specific
3.2	The generation and planning of machine code (fill patterns, build-space orientation, cutting positions, etc.) must be part of the design phase, as the resulting mechanical properties are particularly dependent on the fiber orientation, which should be adapted to the respective load case.	CFR-MEX-specific

**Table 2:** Specification of the manufactured protective mask.

Product Data/Manufacturing Data	Value/Description
Fiber material	Carbon fiber
Matrix material	PA6 (Nylon)
Weight of continuous- fiber-reinforced filament	9.2 g
Weight of thermoplastic filament	83.8 g
Material and production costs per piece	33.80 €
Printing time	~23 h

## Data Availability

The datasets generated and supporting the findings of this article are obtainable from the corresponding author upon reasonable request.
